# Blockchain Framework for Secure COVID-19 Pandemic Data Handling and Protection

**DOI:** 10.1155/2022/7025485

**Published:** 2022-09-14

**Authors:** Arshad Ahmad Dar, Malik Zaib Alam, Adeel Ahmad, Faheem Ahmad Reegu, Saima Ahmed Rahin

**Affiliations:** ^1^Department of Computer Science and Information Technology, Jazan University, Jazan 45142, Saudi Arabia; ^2^United International University, Dhaka, Bangladesh

## Abstract

COVID-19 pandemic caused global epidemic infections, which is one of the most severe infections in human medical history. In the absence of proper medications and vaccines, handling the pandemic has been challenging for governments and major health facilities. Additionally, tracing COVID-19 cases and handling data generated from the pandemic are also extremely challenging. Data privacy access and collection are also a challenge when handling COVID-19 data. Blockchain technology provides various features such as decentralization, anonymity, cryptographic security, smart contracts, and a distributed framework that allows users and entities to handle COVID-19 data better. Since the outbreak has made the moral crisis in the clinical and administrative centers worse than any other that has resulted in the decline in the supply of the exact information, however, it is vital to provide fast and accurate insight into the situation. As a result of all these concerns, this study emphasizes the need for COVID-19 data processing to acquire aspects such as data security, data integrity, real-time data handling, and data management to provide patients with all benefits from which they had been denied owing to misinformation. Hence, the management of COVID-19 data through the use of the blockchain framework is crucial. Therefore, this paper illustrates how blockchain technology can be implemented in the COVID-19 data handling process. The paper also proposes a framework with three main layers: data collection layer; data access and privacy layer; and data storage layer.

## 1. Introduction

### 1.1. The COVID-19 Pandemic

There are a plethora of coronaviruses that can infect people, along with the “novel” one that causes COVID-19. Animals are likely to be here for a considerable time. A virus involving animals can frequently infect humans. Experts claim it is what occurred inside. In other respects, although this virus is not unique to the globe, it is surprising to people. In 2019, when it began to horrify humans, researchers analyzed it as a novel coronavirus. Experts refer to these genotypes as SARS-CoV-2 was the first type known, also called as Alpha-Variant-B.1.1.7 detected in the UK, following the other known type was a Beta variant that was reported first in South Africa, the third variant was gamma reported in Japan, followed by the Delta variant reported in India in late 2021. The currently existing variants are Omicron-B.1.152a caught in South Africa with the new variants known available as on July 2022 include BA.4 and BA.5 variants of COVID-19. Hence, as a consequence of the coronavirus (COVID-19) epidemic that occurred in late 2019, there was an international health crisis. There were more than a million new cases throughout the globe in little over three months, and the virus had spread to various nations. Some of the most affected cities, like Wuhan, New York, Texas, and Hawaii, were reporting new cases by the hour during this period. The number of fatalities also continued to rise due to measures like lockdowns and social isolation, enacted as a response [1].

The cancellation of major international events like the Tokyo Olympics and the Dubai Expo due to COVID-19 has also had a severe effect on the global economy. Therefore, from the first few cases reported by the Globe Health Organization, governments, research centers, and institutions all over the world have been working feverishly to produce a vaccine and forecast the predicted development of the coronavirus.

The daily number of positive and negative cases, tests done, individuals admitted to the hospital, casualties, recorded, space available in ICUs (both empty and occupied), ventilator shortages, and the number of medical experts on-site all play a role in effective containment strategies. COVID-19's development may be monitored and judgments made based on these statistics. As a result, the pandemic of COVID-19 is also known as a data-driven one.

Data availability, verification, and integrity in the COVID-19 pandemic management are crucial for making precise and effective conclusions and recommendations to the public based on records and statistics. For this, tracking applications developed by governments have been adopted to stop the spread of the virus and to ensure the integrity of data. A patient's development following clinical trials may be tracked via tracing applications, making them a key tool for vaccination performance monitoring. Moreover, health facilities use traditional tools such as Excel and centralized databases to store and analyze the data [2].

Governments and significant healthcare facilities have had difficulty managing the pandemic in the absence of appropriate drugs and vaccines. It is also very difficult to track down COVID-19 patients and manage the data produced by the epidemic. Managing COVID-19 data presents additional challenges in terms of data privacy, access, and collecting. With the use of blockchain technology, people and organizations will better manage COVID-19 data thanks to features like decentralization, anonymity, cryptographic security, smart contracts, and a distributed architecture, that is, the miraculous gift of blockchain technology providing the novelty in pandemic data handling and confidential aspects.

### 1.2. Opportunities to Current COVID-19 Data Handling

Blockchain is still a relatively new technology for managing data electronically, but evidently, it has great potential for accountability and supports transparency substantially. A blockchain manages a ledger of transactions, making identical copies for the members to view over the computer network altogether. It implies that all transactions done by the users of the blockchain framework are recorded in some type of database termed as the ledger. Due to its decentralized nature, the similar copies of this information recorded in the above discussed database enable every user to keep the accountability of the electronic data stored in these ledgers. Considerably, blockchain is ideal for transactions that are lightweight in terms of their digital footprint, with the advantages of immutability and greater transparency as well. During COVID-19, the healthcare systems were overwhelmed to a major extent. Blockchain technology plays a crucial role in identity verification, managing the medical supply chain, and with the patient's consent, sharing and accessing patient-specific data throughout the channel [1].

**Figure 1:**
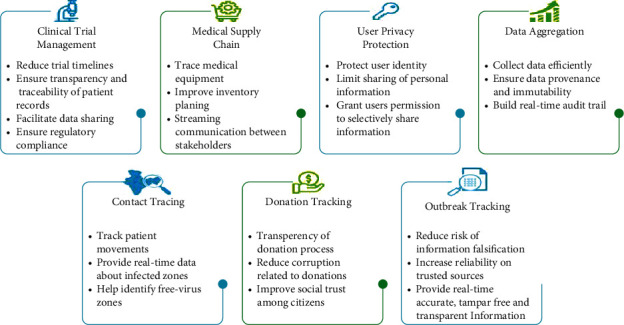
Use of blockchain technology.

Blockchain-enabled tools have emerged to fight the global pandemic in the health sector like an identity management system to support contact tracing in the South Korean region. This system also made the sharing of Covid-specific data available for research purposes as well. Previously, blockchain has been used for supply chain management of pharmaceuticals, for the supply of medicines, adequate medical supplies, and for coming up with appropriate vaccines to combat the deadly virus on the whole [3]. In Figure 1, the potential uses of blockchain technology specific to the healthcare sector are highlighted:

As it turns out, Blockchain is not an ideal option to store high-volume data due to its capacity limitations and on the computational front, it is difficult to replicate data across every network. Moreover, research has found out that storing large records, like electronic medical records or records relating to genetic data, is quite costly over a blockchain and it is inefficient on the whole. It is also very difficult to post query data on a blockchain, which eventually makes the data unavailable for any purpose at all.

Global efforts were made during the pandemic to develop and test vaccinations to stop the viruses' transmission and identify carriers as quickly as possible to find the most effective remedies. Blockchain has the potential to fully satisfy various requirements like security and protection of data, ease of data sharing, and easy access to a vast amount of data as well. During COVID-19, the field of medicine tried taking maximum advantage of blockchain technology through clinical trials and other significant processes involved in the treatment of the virus. The main use of blockchain technology was to manage the epidemic in an effective manner, not only just in the healthcare sector but also in providing adequate solutions for other purposes during lockdowns. Blockchain was evidently very crucial during the COVID-19 times as it offered reliable, transparent, and relatively low-cost solutions to corporations for effective decision-making. The quickness offered through blockchain technology helped many during this crisis and with the passage of time, it is becoming a significant and more integral part of the fight against Coronavirus [4].

The main motive behind the intervention of blockchain technology was to enable easy tracking and effective monitoring, ensuring an adequate supply chain of products and services during COVID-19, and securing the online payments to a major extent as well. All this is possible because blockchain technology is able to create a chronological order of encrypted signatures, a secure ledger that is easily shared by all the members present over the network. However, due to the limitations posed by the infrastructure, on the whole, blockchain technology is not able to fully take advantage of this state-of-the-art unique technology [5].

In comparison with traditional database systems, blockchain technology makes use of its inherent quality to ensure data gathering and management, transactions are transparent, immutable, and accurate due to its decentralized approach, data accessing and storing is much more rapid and does not need the control of central authority as done in the centralized approaches like the DBMS framework where the management transactions are not transparent and require the central control. Blockchain also makes it possible for multiple parties to easily connect in a digitally enabled environment and exchange money without the need for a central authority. Blockchain is transforming a wide range of industries in a variety of ways by easing value exchange and transparency, and boosting confidence across corporate systems. It is used in numerous fields, including finance, legislation, supply chain, travel, and healthcare sector, as it promises to improve data privacy in the healthcare sector and ensure safe data management. As a consequence, it is ideal for dealing with coronavirus-related healthcare issues as well [6].

The COVID-19 outbreak is a knowledge crisis because the available facts are vital in being preventive and protective. However, inaccurate statements that yielded untrustworthy and misleading data greatly expanded the agony of the pandemic. The actual prevalence of the ailment, the proportion of fatalities, indicators, potential therapies, and some of the most effective pandemic control measures have all been distorted by media platforms. The epidemic has aggravated the crisis of conscience in the administrative and medical centers as a result of the collapse to supply of timely and accurate facts about the current predicament. Hence, it leads to the requirements of Covid- 19 data handling to provide all benefits to the user in which they suffered due to misguidance, and therefore to acquire certain features like data security, data integrity, real-time data handling, data management, etc. COVID-19 data handling by the employment of blockchain framework plays a significant role.

This paper highlights the changes that could be made to privacy laws like the Health-Insurance-Portability and Accountability-Act HIPAA to facilitate the seamless and friction-free exchange of patient health information between healthcare organizations that must work together to treat patients as well as the exchange of that information with researchers looking into ways to lessen its effects [7]. The primary aim of the plan is to evaluate the lung computed tomography outcomes of pediatric MPP and MP coupled with streptococcal pneumonia. Clinically, the combination illness of MP and SP is very frequent, and young respiratory doctors must conduct crucial studies on how to diagnose this kind of mixed pneumonia [8]. This paper's method evaluates these photos and summarizes them as positively or negatively for COVID-19 employing existing deep learning models such as VGG19 and U-Net, which have been inspired by new findings that link the occurrence of COVID-19 to findings in Chest X-ray images [9]. In this paper, we construct a deep learning approach to detect COVID-19 using chest X-ray scans and features extracted from the images. ResNet50, InceptionV3, and VGG16 are three prominent models that have been adjusted on an enhanced sample that was constructed by collecting COVID-19 and conventional chest X-ray scans from various database searches [10].

Therefore, from the recent trends available in the study for the detection of COVID-19, we can conclude that before the intervention of blockchain, computed tomography scans, X-ray scans, CNN methods, deep learning methods like ResNet50, VGG19, InceptionV3, VGG16, U-Net, etc., were employed for critical COVID-19 data collection and management.

### 1.3. Challenges with Current COVID-19 Data Handling

However, although the above methods have proven to be effective, various key challenges still limit governments and health institutions from effective containment of patient tracing and developing vaccines for coronavirus.

#### 1.3.1. Data Security Issues

The notion of security encompasses limiting malicious entities from introducing false negatives and false positives into the system and ensuring data integrity. In the context of COVID-19 patient tracing, malicious entities may opt to inject erroneous entries into the data set or cause a denial-of-service attack. These forms of attack are more common with centralized servers, which currently are being implemented by a majority of health facilities.

In most cases, a centralized server is considered secured and trusted. They are responsible for storing a patient's personal identification information (PII) and managing the security keys used to decrypt and encrypt data. However, this poses a great risk to patients' data in case the server is breached. The centralized server acts as a single point of failure and access.

Moreover, centralized servers are concerned with keeping third parties *f* database. In the case that a malicious party is an authorized party on the server, this poses a greater risk to the patient's data since it can easily be leaked, deleted, or altered, resulting in erroneous analysis, statics, and ineffective recommendations to the public [11].

#### 1.3.2. Trust and Data Integrity Issues

The COVID-19 pandemic is an information crisis as the mitigation and preventive measures highly depend on the data available. However, the suffering of COVID-19 has been greatly exacerbated by misinformation leading to unreliable data and inaccurate data. Social media has led to a lot of confusion about the actual prevalence of COVID-19, the number of deaths, symptoms, expected treatments, and some of the best strategies to control the pandemic [12].

Due to the failure to provide timely and accurate data about the current state, the pandemic has worsened the crisis of trust in government and health institutions [13]. Many individuals are looking into other ways to enhance trust within the healthcare system.

#### 1.3.3. Lack of Real-Time Data Recording and Sharing Systems

Global health data synchronization is a key factor to combat the COVID-19 pandemic. Sharing important data such as the number of cases, the positivity rate, the health facilities available, and the progress in clinical trials must occur in real-time to keep the public aware of the current state of the pandemic and to support immediate response to the pandemic.

As mentioned, two particular data sets collected in Wuhan led to different implications due to a mismatch in the number of cases recorded. The first data recorded 425 early cases, while a different data set in Italy and Wuhan recorded a larger number [14].

#### 1.3.4. Isolated and Disintegrated Data Points

To coordinate effective response measures, it is crucial to integrate siloed disparate COVID-19 data types from different nations and health facilities. However, this has been a long-standing challenge in the healthcare industry due to incompatible communication standards in EHR and different jurisdictions in the public sector. The private sector on the other hand brings the challenges of competition and proprietary systems. Therefore, most private health facilities choose to keep the data to themselves to maintain security of their data. While most hospital websites use well-designed websites to display their COVID-19 cases, higher health authorities such as WHO have found it hard to integrate this data or prove its integrity of the data [15].

#### 1.3.5. Data Mismanagement by Intermediary Institutions

Currently, most countries have a pandemic reporting system in which hospitals and clinics diagnose and report to higher national authorities, which in turn report to a higher authority such as the World Health Organization. In cases where there are numerous intermediaries that the records have to pass through the reporting time may result in the loss of some of the data as the records are transferred from one institution to another due to data mismanagement. Furthermore, the use of traditional communication protocols between institutions exposes the data to hacking and security attacks, thus making it more difficult to detect altered data after a hack [16].

#### 1.3.6. Lack of Transparency

COVID-19, like all other pandemics, relies heavily on monetary and in-kind donations to help health facilities and research institutions fight the pandemic. However, throughout the COVID-19 pandemic in 2020, numerous corruption cases have been reported [17]. This can largely be attributed to the centralization and “black-box” aspect of the current logistics, warehousing, expenditure, and distribution of funds in health facilities.

Therefore, innovative technologies like Artificial Intelligence, Internet of things, and blockchain can be used to alleviate some of these issues. Blockchain in particular provides key features that would revolutionize how governments and health institutions handle COVID-19 data. However, there are security concerns with respect to blockchain technology such as data vulnerabilities and double-spending activities present in blockchain consensus protocols. And yet, there are still a variety of challenges like legal issues, latency issues, resource utilization issues, and widespread implementation issues that have to be resolved before the blockchain can be fully implanted to counter COVID-19 and enhance COVID-19 preventive measures.

### 1.4. Contribution

Blockchain technology is a decentralized technology with an immutable information structure, transparency, and enhanced security through cryptographic encryption. It is a decentralized ledger technology, that is, distributed and stores data in blocks chained together by hash functions [18].

#### 1.4.1. Features of Blockchain Technology

*(1) Decentralization*. The technology offers decentralization by dis-intermediation of central entities and offers equal opportunity in decision-making to all peer nodes in the network.

*(2) Consensus Mechanism*. It is a fault-tolerant decision-making mechanism that allows peer nodes to reach a conclusion fairly on a network.

*(3) Distributed Ledger*. Any transaction data recorded on the blockchain is distributed to all authorized peer nodes. Each node can view and verify the authenticity of the data and its source.

*(4) Immutable and Tamperproof Ledger*. Once a transaction is committed to the blockchain, it is almost impossible to alter the data. Moreover, any alterations to the data will have to be agreed upon by at least 50% of the nodes in the network. This leads to tamper-proofing of transactions recorded on the blockchain.

*(5) Smart Contracts*. A smart contract is a self-executing piece of computer code that encodes the terms and conditions of an agreement in a legal agreement within the code. Any node on the blockchain network can initiate the smart contract if certain preset conditions are met [19].

This paper looks at how the key features of blockchain technology can be implemented in the healthcare industry to allow for secure and efficient data handling of COVID-19 data.  Table 1 shows the comparison between the traditional client-centered centralized platform and blockchain platforms.

#### 1.4.2. Layout of the Present Study

The various modules of this paper are organized as follows: Section 1 contains an introduction followed by Section 2 which details some of the use cases of blockchain technology for COVID-19, Section 3 proposes a framework that can be implemented to allow secure data handling with regards to the COVID-19 pandemic, Section 4 details some of the expected challenges in implementing blockchain technology, and Section 5 presents the conclusions of the paper.

## 2. Use Cases of COVID-19 Based on Blockchain

Improved data sharing, patient tracking, and clinical trials might all benefit from the adoption of blockchain technology in the healthcare sectors impacted by the coronavirus epidemic. An in-depth analysis of the existing research on the potential use of blockchain technology in the battle against COVID-19 may be found in this section.

### 2.1. Contact Tracing

COVID-19 has an average incubation period from infection to symptoms of about 5.5 days, and it is also estimated that most cases are symptomatic. Therefore, keeping track of the current positive cases and who they get in contact with is very crucial. Governments and health care facilities have been using mobile apps to trace patients, but there are still some existing challenges as mentioned above.

By enhancing the quality and dependability of data collected via mobile apps, blockchain technology adds an extra degree of security. Since blockchain has a write and read model, the data collected from patients is immutable. Blockchain technology also monitors patients' conditions, virus-prone areas, and safe areas and updates this information to the distributed ledger in real-time. This ensures that government and health institutions are aware of the current state of an area at a particular time.

To offer these services, blockchain technology also incorporates other technologies such as AI, IoMT, and geographic information system. It means blockchain technology employs read-write model to keep track of data gathering and patient monitoring, and it can also assist us in obtaining the information related to areas that prevail under the safe zone or virus zone and hence keep the distributed ledger to update all these details. Moreover, it not employs blockchain technology as the fundamental base but also unites other methodologies like AI, IoMT, and GIS for enabling us with these features. There, blockchain technology provides a practical approach to tracing COVID-19 cases and provides a reference point for making future containment decisions and recommendations [20].

### 2.2. COVID-19 Database Integration

Data collection, accumulation, access, and information exchange among health institutions is a major opportunity for a worldwide response to the epidemic. Blockchain technology provides the opportunity to ascertain data integrity through its immutable features. The architecture of blockchain acts as a single ledger that can be implemented in the healthcare sector to record COVID-19 data. Any data collected from testing and clinical trial data is uploaded and stored on the blockchain. Authorized researchers and institutions can access the data and utilize it to further their research towards developing a cure.

Blockchain also provides four key frameworks, public blockchain, private blockchain, consortium blockchain, and hybrid blockchain [21]. In the above discussed frameworks, the first one is entirely distributed, with no requirement of permission, and available to everyone who joins. Blockchain systems enable all nodes to still have identical access, the opportunity to add new data items, and the potential to certify existing chunks of data. The second one is the restricted blockchain systems, also known as administered blockchains, which are governed by a singular entity. Who is permitted to be a node on a permissioned blockchain is regulated by the central authority. Additionally, the central body may not always accord every node an equal right to execute certain duties. Instead of being regulated by a specific organization, as with a private blockchain consortium, blockchains are permissioned blockchains overseen by the group. As a result, this framework of blockchains is more global than private blockchains, which boosts their resilience. Blockchain systems that are managed by a singular body but have some supervision offered by public blockchains, which would be necessary to carry out specific transaction approvals, are known as hybrid blockchains. Besides public blockchain, the later frameworks empower compliance management and audibility by providing digital keys to grand flexible and secure data sharing through different managerial levels.

### 2.3. Clinical Trial Data Management

Clinical trials are very crucial when developing a cure to the coronavirus pandemic. However, every product should be thoroughly tested to ensure that it matches the required standards and note the possible side effects of the products. Clinical trials are carried out in three phases, of which the third phase incorporates the largest number of test participants, which makes it a challenging and resource-intensive phase.

Blockchain technology helps ensure the safety and privacy of the data. Additionally, since it has a distributed framework, the technology distributes the resources required to manage the network to various health institutions. As a result, this encourages trial data sharing and ensures regulatory compliance within the healthcare facilities [22]. It also creates a continuous and transparent medical record that allows researchers access to the trial data at any point in the trial process.

### 2.4. User Privacy Protection

Health care facilities and government agencies are finding it more difficult to strike a balance between the need to gather enormous amounts of data and the need to protect patient privacy. Data may be collected and stored in a more secure manner using blockchain technology. Within a blockchain network, patients are provided with unique identification numbers known as addresses. Blockchain addresses are anonymous and cannot be linked back to the patient's personal information or identification. However, any information transaction initiated by a patient can be easily verified on the blockchain using Merkle trees [23].

The user's privacy protection in the blockchain network is acquired with the assistance of secret keys. The usage of encryption keys is really a crucial component of privacy protection in blockchain technology. Asymmetric encryption is being used by blockchain networks to authenticate client interactions. Every client on these platforms has a pair of keys, essentially a public/private key, respectively. These keys are interconnected cryptographically and comprised of random data. A user can never logically extract another participant's secret key from their public key pair. Clients are guarded against hackers and protection is improved dramatically [24].

As a result, COVID-19 patients can easily upload their records to the blockchain or even share them publicly without the fear of exposing their identity.

### 2.5. Secure Data Storage and Sharing

One of the key advantages of blockchain technology is providing verifiable and secure data by using its distributed and decentralized features. During the COVID-19 pandemic, blockchain is instrumental in recording and storing the patient's data such as test results, symptoms, location, and medical history of the patient in a secure and immutable manner.

## 3. Proposed Blockchain Framework

**Figure 2:**
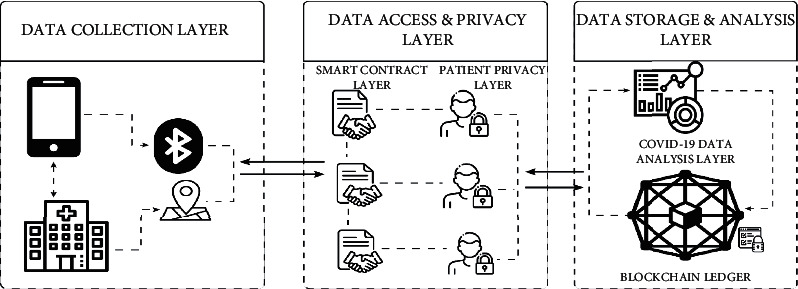
Proposed blockchain framework that can be implemented on COVID-19 data handling.

In this section, the paper proposes a framework with four layers in the system and the interactions within the data handling process within the system.  Figure 2, labeled as “proposed blockchain framework that can be implemented with COVID-19 data handling” depicts the main three layers under the headings of data collection: which aggregates the data from smartphones, hospital devices, and other data assembling equipment's, data access and privacy layer: it is a supporting element of the framework since it regulates whom or how can we access the data. Any data included in smart contracts is timestamped, and the location is likewise logged and data storage analysis: the patient data in this architecture is kept in decentralized databases that are contributed by various hospital nodes and user devices. Active medical equipment serves as a peer-to-peer node and provides data storage. All these modules are further detailed in-depth under Sections 3.1, 3.2, and 3.3, respectively.

### 3.1. Data Collection Layer

#### 3.1.1. User Interface Layer

At the user interface layer, there are three components, smartphones, hospital devices, and GIS system. Within this layer, the system collects the patients' COVID-19 status and their geographic location. The patient's data were recorded in two states, user positive or user negative. The geographic location of the patient is the health facility or clinic at which the patient was tested. The framework requires two data entry points to maintain the honesty of a patient's record. The first data entry point is from the patient's smartphone while the second entry is from the health facility. The two data points have to be consistent; otherwise, they will be rejected by the smart contract layer [16].

#### 3.1.2. Mobile Service Layer

**Figure 3:**
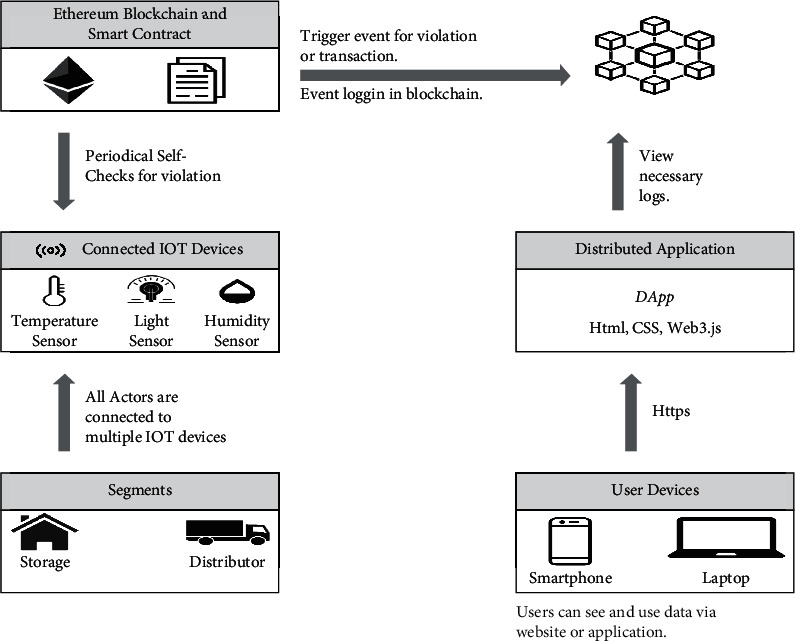
DAPPS Layer that enables the users to access information on their smart devices.

The mobile service layer is a core component of the framework as it hosts decentralized applications (DAPPS). DAPPS layer contains decentralized applications that collect data from the patients and interacts with the smart contract layer. The mobile service layer provides users and health facilities with crucial services: contact tracing based on Bluetooth, GIS systems, and health tracking services supported by data from the blockchain data storage layer [1]. In Figure 3, you can see the DAPPS Layer that enables users to access information on their smart devices.

### 3.2. Data Access and Privacy Layer: Smart Contracts

The smart contract layer is the secondary component of the framework as it governs how and who accesses the data. Any record integrated into the smart contracts was time-stamped and the geographic location is also recorded from the highest level, country level to the smallest level, state level (depending on the country). Each contract will inherit data from a lower level contract, but two similar data points from a single patient will not belong to a single superior contract [25].

Moreover, data access will be restricted based on permissioned blockchain. The framework will provide various networks for users and the hospital to utilize. Within the network, numerous channels will be created to allow secure data sharing. The smart contracts will also implement unique identifiers for the patients to maintain their identity. Any patient's data stored will be recorded as follows: location status, infection status, and think progress. The contract will also be active for 14 days after the patient tests positive. Only after 14 days will patient data be considered clean status [26].

To save the operation cost of data storage, the patient data are hashed and only the head of the block is stored within user devices, the rest of the data is stored on the data storage layer.

### 3.3. Data Storage and Analysis Layer

Within the proposed framework, the patient's data is stored within decentralized databases provided by different hospital nodes and user devices. Active hospital devices act as peer-to-peer nodes that provide storage for the data. The active nodes are incentivized to participate in the network by being allowed access to blockhead data. Since Bitcoin and Ethereum blockchain database designs have high computational costs and slow transaction speed. Moreover, it is a peer-to-peer (P2P) network that can be used to transmit Bitcoin, a virtual currency, without the necessity for centralized power. In 2008, somebody or a body of citizens named Satoshi Nakamoto designed it. An irreversible decentralized system contains all transactions. On the other hand, blockchain-based shared platform Ethereum. Where ether is the name of Ethereum's network currency, the transactions in this instance are also kept in an immutable decentralized ledger. Therefore, the Hyperledger blockchain can be implemented in the framework to provide a hybrid blockchain platform as shown in Figure 3.

### 3.4. Effective Uses of Blockchain Technology during COVID-19

When COVID-19 had hit the world in the year 2019 and took a stroll all over the world in the following year, the country struggled to gain control over its spread, be it the first world nations or third world nations. The countries were on the lookout for innovative and quick technologies that could help in managing the large amounts of data accumulated with respect to the Covid virus. The healthcare sector was one of the primary sectors to have been affected by this pandemic the most as it had no experience in dealing with such viruses ever before. Where at one end the healthcare sector was dealing with the large influx of patients, it had to be clinical trials to get to the root cause of the virus and come up with an effective remedy for it. In order for the clinical trials to run smoothly, it required a management system that was transparent and fair by all means. The countries were clear about the priorities of this system it had to be cost-effective since already the healthcare sector was facing tremendous expenses due to this global pandemic. Moreover, it was required to be fast, transparent, auditable, and compliant with the regulations everywhere. The following section will give a detailed overview of the application of blockchain technology in the form of effective solutions within the healthcare sector to gain control over the spread of COVID-19 [27].

#### 3.4.1. Clinical Trial Management

The main advantage of incorporating blockchain technology into this system was that it enables medical practitioners and researchers to gain access to clinical data in a timely manner with greater accuracy altogether. One such application was introduced by the Canadians where it used blockchain technology to assist the local authorities and help the government bodies to a major extent in gaining control over the pandemic. Moreover, this application is also facilitated in clinical trials in relation to COVID-19, it associates the data with the individual person's ID, but the corresponding blockchain also records anonymous data inputs ensuring that the patient's sensitive information is not easily available to all. This technology can help in keeping track of the infected patients based on the data in the application this was a significant step toward ensuring that the spread of the virus was minimized to a major extent. It can further enable doctors to track the overall progress of the patients if they are infected by a coronavirus. The doctors can interact and share medication procedures to help out patients, who have isolated themselves at home. However, this application has some sensitive points that can question the sanctity and genuineness of the data shared on this blockchain technology [28].

#### 3.4.2. User Privacy Protection

During COVID-19, where all sectors were posed with major challenges left, right, and center, there was a dire need to strike a balance and that was possible through data collection and assurance of privacy to the end-users. Blockchain can be effectively used in collecting significant pieces of information with respect to examination of the patient and be able to screen the movement of the patients to make sure that they are adhering to the SOPs of COVID-19, taking social distancing measures seriously. Nonetheless, protection of the patient's identity was essential all this while. Since there is no central authority to speak of with blockchain technology, users have complete control over the data they send and receive through the blockchain network. Blockchain technology may be used by healthcare organizations while maintaining a strict emphasis on safeguarding patient privacy and identification at all costs. A similar framework was introduced in Europe, where blockchain technology was used in COVID-19 tracing by making use of Bluetooth. On the other, a German company has made use of a blockchain framework using cellphones, while making sure that the client's security is not violated in any manner [29].

#### 3.4.3. Managing Supply Chain

With the pandemic hitting the entire world, there were some significant interruptions across supply chains in all different sectors. The primary factors that were responsible for these interruptions were the shutting down of factories and improper or inadequate safety and hygiene measures followed in the facilities altogether. There was also an enormous demand for PPE kits and other important medical supplies to support the production and continuation of operations in corporations everywhere. Due to the lengthy supply chain, it was resulting in excess obscurity that made it more difficult to track, calculate, and plan the supply processes altogether. Blockchain also ensures that the network is transparent and the transactions are securely broken down for further processing. As a result, large numbers of blockchain arrangements were incorporated into the supply chain during the pandemic. The advantages were substantial, it not only made it fairly quick to handle and process but also reduce the overall costs, low risk of operations, and faster settlement for all included within the network. There is a blockchain platform by the name VeChain, that is, ensuring a sustainable yet transparent supply of new KN95 masks from China while working with numerous production facilities and different corporations.

#### 3.4.4. Outbreak Tracking

Amidst the outbreak of COVID-19, it was the most difficult task for the government to identify the infected patients and then get them to be secluded from the rest to gain control over the wide spread of the virus altogether. Blockchain removes the potential need for any kind of third party since it is highly decentralized that can potentially reduce the occurrence of data modification to a major extent. It is due to the use of blockchain technology that has potentially enhanced the reliability of the information available on the pandemic for the population in general. Evidently, fraudulent data and false information not only contribute to the enormous damage made to the economy as a whole but also end up affecting the psychological state of the general population altogether. Therefore, a blockchain database is highly reliable in such a vulnerable state of events, where it probably makes it more difficult to modify or make the data nontraceable on the whole.

Because of the accuracy, reliability, and transparency that blockchain technology provides, it is an ideal tracking platform. Governments can better update their databases as well with respect to coronavirus' current status and further improve the planning and management of the outbreak as well. This technology can also help in identifying the territories that are completely no-go zones and further tracking down the spread of the infection altogether. Hash-Log, a public database maintained by the prominent IT company Acer, provides information on the propagation of infections and allows users to follow the patterns over a certain period of time. For clinical studies, the Acer dashboard additionally makes use of data from the Centers for Disease Control and Prevention and the World Health Organization (WHO).

## 4. Conclusion

COVID-19 pandemic has affected numerous sectors of day-to--to-day life like healthcare, education, politics, and the economy to mention a few. Blockchain technology plays a major role in the management and development of preventive measures. Because the epidemic has aggravated the crisis of conscience in the administrative and medical centers as a result of the collapse to supply of timely and accurate facts about the current predicament. Hence, it leads to the requirements of Covid- 19 data handling to provide all benefits to the user in which they suffered due to misguidance, and therefore to acquire certain features like data security, data integrity, real-time data handling data management, etc. COVID-19 data handling by the employment of blockchain framework plays a significant role. Hence, in this paper, the key features of blockchain technology have the potential to support the implementation of many use cases such as contact tracing, efficient data collection, secure data storage, and effective data analysis. Comparing Blockchain data handling systems to centralized database systems, blockchain provides the potential to improve COVID-19 Data handling to a major extent.

Before the blockchain can be fully implemented to combat COVID-19 and improve COVID-19 preventive measures, a number of problems, including legal issues, latency issues, resource usage issues, and widespread implementation issues, must be handled. We put out a number of potential options for enhancing blockchain adoption in the future in light of the COVID-19 outbreak. By offering a secure framework for capturing, maintaining, and sending sensitive data, blockchain applications may be utilized to decrease network latency. Furthermore, COVID-19 management will be made possible by the recent integration of blockchain with other cutting-edge technologies including artificial intelligence, big data, and cloud computing. It is also suggested that blockchain is being coupled with other current technologies to give an adequate performance in resolving issues related to the COVID-19 pandemic to build a strong healthcare infrastructure. Furthermore, by utilizing new cutting-edge and distinctive security methodologies, the corresponding security risk regarding blockchain technology can be addressed. To address data vulnerabilities in blockchain consensus protocols, security defense solutions are gathered under this heading. A recipient-oriented transaction plan that uses the phrases “stealth address and master node” to authorize the transaction before adding it to the block is presented as a solution to the problem of double spending in blockchain. In this case, both the transaction's originator and the recipient actively take part in the broadcast process's transaction verification. The patient's data can be made more secure and protected overall by implementing this feature in the healthcare industry. Finally, the blockchain-based application needs to be upgraded to outperform in several technological aspects with the aim of employing the blockchain as a model alternative for the crisis in the healthcare domain, akin to the COVID-19 pandemic. Scalability and lightweight blockchain implementation, for instance, are essential in the healthcare industry to minimize data verification and delay. To minimize processing time and enable high-speed data sharing, effective mining techniques may be utilized in conjunction with shorter verification operation times, leading to robust data analysis in a setting identical to the COVID-19 environment.

## Figures and Tables

**Table 1:** Comparison between traditional client-centered centralized platform and blockchain platform.

Aspect	Traditional platforms	Blockchain platform
Data handling	Supports read, write, amend, delete and update operations	Only allows read and write operations
Integrity of data	It's possible to tamper with or change data.	Data is immutable and easily auditable
Privacy of data	Highly susceptible to cyber-attacks and PII leaks	Data is stored using cryptographic hash functions that make PII anonymous
Transparency of data	Databases are not transparent or easily auditable	Data is available and stored in distributed ledger
Data security	Single-point failures or assaults provide a high risk.	Distributed ledger hence highly fault-tolerant

## Data Availability

The data shall be made available on request.
